# Prevalence and determinants of hepatitis C virus infection among female drug injecting sex workers in Glasgow

**DOI:** 10.1186/1477-7517-5-11

**Published:** 2008-03-20

**Authors:** Avril Taylor, Sharon J Hutchinson, Gail Gilchrist, Sheila Cameron, Susan Carr, David J Goldberg

**Affiliations:** 1 Institute for Applied Social and Health Research, School of Social Sciences, University of the West of Scotland, Paisley Campus, Paisley PA1 2BE, UK; 2 BBV/STI Section, Health Protection Scotland, Clifton House, Clifton Place, Glasgow, G3 7LN, UK; 3 Addiction and Substance-Related Disorder Research Group, Institut Municipal d'Investigació Mèdica (IMIM)-Hospital del Mar, Parc de Recerca Biomèdica de Barcelona, c/Dr. Aiguader, 88, Barcelona, 08003, Spain; 4 West of Scotland Specialist Virology Centre, Gartnavel General Hospital, Glasgow, G12 OYN, UK; 5 Family Planning & Reproductive Health, The Sandyford Initiative, 2-6 Sandyford Place, Sauchiehall Street, Glasgow, G3 7NB, UK

## Abstract

**Background:**

Few studies of the prevalence of hepatitis C virus (HCV) infection have focussed on women who work as street sex workers to finance their drug use.

**Methods:**

The investigators report the survey findings of such a population in Glasgow. All women attending the health and social care drop-in centre, situated in Glasgow's "Red Light Area", during a four-week period in 1999 were invited to participate in a survey involving the provision of a saliva sample for anonymous HCV testing and the self-completion of a questionnaire seeking demographic, sexual and injecting practice data.

**Results:**

Of the 223 women who attended, 51% agreed to participate. Of the 98 women who provided a sufficient saliva sample, 64% (95% CI: 54%–74%) tested HCV antibody positive; 98% of those who tested positive had ever injected drugs. Adjusting for the 85% sensitivity of the saliva test, the HCV antibody prevalence among IDU sex workers sampled was 81%; a rate which is considerably higher than those recorded, contemporaneously, among Glasgow IDUs generally. Two factors were independently associated with HCV antibody positivity in saliva: ever shared needles and syringes (adjusted OR 5.7, 95% CI 2–16) and number of times imprisoned (adjusted OR 7.3, 95% CI 1.4–39, for more than five times compared to zero times).

**Conclusion:**

Women who engage in street sex work to finance their drug habit are a particularly desperate, chaotic and vulnerable population. This study demonstrates that their HCV infection risk may be greater than that for other IDUs. Those responsible for designing interventions to prevent HCV infection among IDUs should consider the special needs of this group.

## Background

Hepatitis C Virus (HCV) infection is one of the major public health issues of the 21st Century. In the UK, it is estimated that 200,000 to 450,000 individuals, mostly persons who have injected drugs, are infected with HCV [1,2]. Many studies of the prevalence and determinants of HCV among injecting drug users (IDUs) have been undertaken [3] but few have focussed on women who work as street prostitutes to finance their drug habit [4]; previous studies among female sex workers have generally involved a low proportion (≤ 5%) of IDUs [5,6]. The investigators report the survey findings of such a population in Glasgow, a city with one of the highest prevalences of injecting drug use in Europe (2.7% of 20–29 year olds in 1990) [7].

## Methods

### Setting and Population

The study was conducted among street sex workers (i.e. sex workers who solicited clients from the street) attending a health and social care drop-in centre (DIC) for sex workers, established in 1988 in Glasgow's "Red Light Area" [8]. The centre is open nightly, except Saturday, from 7.30 pm until midnight and is managed jointly by the Greater Glasgow Health Board and the Glasgow City Council Social Work Department. It provides a social environment where women can chat, have tea and sandwiches and use a wash-room. Social Work staff provide information, advice and organisational support, and a doctor and a nurse run a medical service which responds to a wide range of conditions, particularly those relating to drug-injecting. Condoms are freely available, as are needles and syringes. One thousand and three different street sex workers had attended the DIC between January 1995 and March 1999.

### Design

All women attending the DIC during a four-week period in 1999 were invited to participate in a survey involving the provision of a saliva sample for anonymous HCV testing and the self-completion (overseen by a researcher) of a questionnaire seeking demographic, sexual and injecting practice data.

### Saliva testing of HCV antibodies

A modified ELISA assay (Monolisa anti-HCV-Sanofi Pasteur, France) [9] was used by the West of Scotland Regional Virus Laboratory, Gartnavel General Hospital, Glasgow, to detect HCV antibodies in saliva; the assay's sensitivity and specificity were 85% and 100%, respectively [9].

### Data Analysis

All analyses were conducted using S-PLUS Software [10]. Univariate and multifactorial logistic regression analysis was performed to determine the main predictors of an HCV antibody test result in saliva; all factors significantly associated with HCV antibody status at the 5% level in the univariate analyses were considered in the multiple regression analyses.

## Results

### Sample Characteristics

Two hundred and twenty three women attended the DIC during the study period; 114 (51%) agreed to participate. All non-participants gave their reasons for non-participation as "insufficient time" or "not interested". The mean age of participants was 26 years (range 16–46); 19% (21/111) were aged less than 21 years. The mean age of commencement in street sex work was 21 years (range 10–36).

Respondents had worked as street sex workers for a mean of 4.6 years (range 0–32; SD 5.9) and had been attending the DIC for a mean of 3.5 years (range 0–12; SD 3.3). Seventy-three per cent (81/111) had begun using the DIC in the year they began street sex working.

Ninety-three per cent of women (99/107) had ever injected drugs. Eighty four percent (96/114) of all respondents and 93% (92/99) of those who had ever injected reported that they were currently taking heroin. Most IDUs had commenced injecting prior to, or in the year they commenced street sex work.

### Risk Behaviour

Of the 99 respondents who had ever injected drugs, 84% (83/99) were current IDUs, injecting, on average, two to three times daily. Ninety-seven per cent (100/103) and 90% (96/107) of women reported using condoms "all of the time" when having straight and oral sex with their clients, respectively.

### Hepatitis C Saliva Test Results

Saliva samples were obtained from 103 participants; 5% of these proved insufficient for testing. Of the remaining 98, 63 (64.3%, 95% CI: 53.9%–73.5%) tested HCV antibody positive.

Ninety-eight per cent (61/62) of those who tested positive had ever injected drugs; the injector status of one HCV positive individual was not reported. Of those who provided a sufficient sample and had ever injected, 68.5% (61/89) were HCV antibody positive in saliva. Among non-injecting sex workers, the prevalence of HCV antibodies was 14.3% (1/7).

### Determinants of HCV antibody positivity in saliva (Table 1)

**Table 1:** Logistic risk scores for hepatitis C antibody positive status in saliva (n = 98 female street sex-workers)

**Determinant**		**Total**	**Hepatitis C antibodies in saliva**	**Odds Ratio (95% confidence interval)**^‡^	
				
		N (%)	n (% of N)	Univariate	Multifactorial
Study group		98 (100%)	63 (64%)		
Age (years) (3 non-responses)	16–20	18 (19%)	7 (39%)		
	21–25	34 (36%)	21 (62%)	1.08 (0.99–1.17)	NS
	26–30	29 (31%)	25 (86%)		
	> 30	14 (15%)	9 (64%)		
Time since began prostitution (years) (2 non-responses)	≤ 1	40 (42%)	23 (58%)	1.00 (Baseline)	
	2–5	32 (33%)	22 (69%)	1.63 (0.61–4.31)	NS
	> 5	24 (25%)	17 (71%)	1.80 (0.61–5.29)	
Time since onset of injecting drug use (years) (2 non-responses)	Never injected	7 (7%)	1 (14%)		
	< 2	30 (31%)	15 (50%)		
	2–5	27 (28%)	18 (67%)	1.20 (1.06–1.35)*	NS
	6–10	19 (20%)	16 (84%)		
	> 10	13 (14%)	12 (92%)		
Ever shared needles for injecting (4 non-responses)	Yes	47 (50%)	40 (85%)	**6.31 (2.31–17.29)**	**5.75 (2.01–16.46)**
	No	40 (43%)	19 (48%)	1.00 (Baseline)	1.00 (Baseline)
	Never injected	7 (7%)	1 (14%)	0.18 (0.02–1.63)	0.26 (0.03–2.48)
Ever received help for drug problems (3 non-responses)	Yes	68 (72%)	49 (72%)	**3.22 (1.28–8.13)**	NS
	No	27 (28%)	12 (44%)	1.00 (Baseline)	
Number of times in prison (16 non-responses)	None	33 (40%)	16 (49%)	1.00 (Baseline)	1.00 (Baseline)
	1–5 times	26 (32%)	14 (54%)	1.24 (0.44–3.47)	1.27 (0.39–4.14)
	> 5 times	23 (28%)	21 (91%)	**11.16 (2.26–55.16)**	**7.30 (1.35–39.46)**

‡ Results in bold are statistically significant at the 5% level.

* Time since onset of injecting drug use was entered as a continuous variable in the regression analysis, where non-injectors were coded as missing values.

NS indicates not included in the multifactorial model, because of lack of significance.

At a univariate level, respondents who had been injecting for more years were significantly more likely to be HCV antibody positive; the prevalence of HCV antibodies in the saliva of those who had injected for under two years was 50%, while that for more than ten years was 92%. Respondents who had ever received help for their drug problem were also significantly more likely to test HCV antibody positive in saliva (with a prevalence of 72% compared to 44% among those who had never received help for their drug problem). Respondents who had ever shared needles were significantly more likely to be HCV antibody positive in saliva (85% compared to 48% among injectors who had never shared) and those who had been incarcerated for more than five times were significantly more likely to be HCV antibody positive in saliva (91% compared to 49% among those who had never been imprisoned).

In the multiple regression analysis, two factors were independently associated with HCV antibody positivity in saliva: i) ever shared needles and syringes (adjusted OR 5.7 (95% CI 2–16), compared to IDUs who reported never having shared) and, ii) number of times imprisoned (adjusted OR 7.3 (95% CI 1.4–39) for more than five times, compared to zero times). After adjustment for these two factors, other variables, including those significant at a univariate level (i.e. time since onset of injecting or ever received help for drug problems), were not significantly associated with HCV antibody status and were excluded from the final multiple regression model shown in Table 1.

## Discussion

The sample was not necessarily representative of Glasgow's street sex workers due to potential participation bias as a consequence of refusal to be studied, and selection bias due to the approach of convenience sampling in a health and social care setting for this population. Nevertheless, the finding that nearly three-quarters of respondents had attended the DIC within one year of beginning street sex work is consistent with a very high proportion of Glasgow's city centre street-workers accessing this service; further, the average number of women attending the DIC during the 1990s was 500–1,000, a figure similar to the estimated number of women working as street sex workers in Glasgow at any one time [11].

Adjusting for the 85% sensitivity of the saliva test, the HCV antibody prevalence among IDU sex workers was 81%, a rate which is considerably higher than those (60–70%) recorded, contemporaneously, for Glasgow IDUs generally [1,12]. Indeed, this prevalence is considerably higher than any recorded among IDU populations throughout the UK between 1991 and 2005 [13].

The HCV antibody prevalence of 50% among sex workers who began to inject in the previous two years – between 1997 and 1999 – indicates that harm reduction interventions, such as needle/syringe exchange and methadone maintenance treatment – introduced in the late 1980s and early 1990s – had not controlled the spread of HCV in this population. This rate was significantly (χ^2^(1) = 10.3, p = 0.001) higher than that found in a community-wide sample of IDUs, recruited in Glasgow during 1999, who had injected for under two years (20% in saliva, 27/136; unpublished data, Health Protection Scotland). Despite the provision of a wide range of services at the DIC, including guidance about safe injecting, counselling and needle and syringe provision, and that the DIC was attended by a large proportion of participants within 12 months of their injecting and sex working debuts, the incidence of HCV infection among this group remains high. Research has shown that while harm reduction programmes (e.g. methadone maintenance treatment and needle exchange programmes) have been effective in reducing the incidence of HIV [14,15], they have been less successful in reducing the incidence of HCV [16-19]. Some authors have suggested this may be the result of the harm reduction effort focusing on needle and syringe sharing [20] when the sharing of injecting paraphernalia other than needles and syringes (e.g. spoons and filters) has been demonstrated to increase HCV transmission [21-23]. However, data on paraphernalia was not collected in the current study.

The two principal determinants of HCV antibody positivity – ever sharing needles/syringes and frequency of imprisonment – have been widely recognised among IDUs [19,24,25], though, hitherto, not specifically in this sub-group.

The high rate of HCV antibody positivity (48%) among the IDUs who declared never sharing needles and syringes is not an uncommon finding. Poor recall, mendacity and the acquisition of HCV through, for example, the sharing of other injecting equipment such as spoons and filters [26] are possible reasons for this observation.

Since the women reported that they almost always used condoms when engaging in sexual intercourse with their clients and since the efficacy of HCV transmission through such unprotected behaviour is known to be low, appreciable sexual transmission of infection between such sex workers and their clients is unlikely to occur. HCV prevalence surveys of non-IDU sex workers, the numbers of whom were considerably greater than that recruited in this investigation, indicate rates of less than 1% [4,27-29].

## Conclusion

This study examines the prevalence and determinants of hepatitis C virus infection among female drug injecting sex workers, an understudied and difficult to reach population. Women who engage in street sex work to finance their drug use are a particularly desperate, chaotic and vulnerable population. The HCV antibody prevalence among IDU sex workers sampled was 81%. Therefore, this study demonstrates that their HCV infection risk may be greater than that for other IDUs. Furthermore, the majority of those interviewed reported using condoms with clients highlighting the effectiveness of the DIC in addressing sexual risk taking behaviour among sex workers. Therefore, it is unlikely that the increased prevalence is a result of sexual transmission, rather the results demonstrate that ever sharing needles and syringes and the frequency of incarceration were associated with HCV antibody positivity. Thus, those responsible for designing interventions to prevent HCV infection among IDUs should consider the special needs of this group. Since 1999, a methadone clinic has been operational at the DIC and a needle exchange service established in hostels for homeless persons – many of whom are IDU sex workers. It remains to be seen how effective these additional initiatives have been in stemming the spread of HCV among this population.

## Competing interests

The authors declare that they have no competing interests.

## Authors' contributions

Avril Taylor conceived of the study, participated in its design, project managed the study and drafted and critically revised the manuscript. Sharon Hutchinson performed the statistical analysis, and drafted and critically revised the manuscript. Gail Gilchrist participated in the study design, collected data and samples, and drafted early manuscript. Sheila Cameron conducted analysis of saliva samples. Susan Carr conceived of the study, and participated in its design. David Goldberg conceived of the study, participated in its design and critically revised the manuscript. All authors read and approved the final manuscript.
